# Effect of advanced pregnancy on intraocular pressure, tear secretion and hemogram values in Holstein cows

**DOI:** 10.1007/s11250-026-04957-3

**Published:** 2026-03-02

**Authors:** Tuba Özge Yaşar, Kudret Yenilmez

**Affiliations:** 1 Department of Surgery, Faculty of Veterinary Medicine, Tekirdağ Namık Kemal University, Tekirdağ, Türkiye; 2 Department of Obstetrics and Gynecology, Faculty of Veterinary Medicine, Tekirdağ Namık Kemal University, Tekirdağ, Türkiye

**Keywords:** Intraocular pressure, Schirmer tear test, Pregnancy, Hematological parameters, Holstein cows

## Abstract

This study investigates how pregnancy influences ocular physiology and hematological parameters in Holstein cows as a topic with limited representation in veterinary research. Thirty clinically healthy cows, aged 2–6 years, were examined and categorized into pregnant (*n* = 15) and non-pregnant (*n* = 15) groups. Intraocular pressure (IOP) and tear production (Schirmer Tear Test, STT) were measured bilaterally, and comprehensive blood profiles were evaluated. No statistically significant differences were observed between groups in IOP and mean STT values (*p* > 0.05); however, a notable asymmetry in tear production was found between the right and left eyes of pregnant cows (*p* < 0.05), suggesting a localized physiological modulation during gestation. Hematological results revealed significant changes in mean platelet volume (MPV) and atypical lymphocyte percentages and counts (%ALY, ALY) (*p* < 0.05), highlighting the systemic immune and hematologic adaptations that accompany pregnancy. These findings underscore the importance of recognizing pregnancy-induced physiological shifts in clinical evaluations to avoid misinterpretation of normal adaptive changes as pathological. Future research integrating hormonal profiling could provide deeper insight into the mechanisms driving these alterations.

**Supplementary Information:**

The online version contains supplementary material available at https://doi.org/10.1007/s11250-026-04957-3.

## Introduction

Intraocular pressure (IOP) values can be influenced by various factors, including the type of measurement device used, the experience of the examiner, the species and breed of the animal, stress levels, the time of day the measurement is taken, and environmental conditions (Cahacaltana et al. 2016; Ghaffari et al. 2011). During gestation, multiple organ systems undergo adaptive physiological modifications (Soma-Pillay et al. 2016). The eye is among the organs affected by these physiological changes during pregnancy. Human studies indicate that gestation is associated with widespread ocular alterations, suggesting that pregnancy-related systemic changes extend to visual structurespregnancy, including the eyelids, cornea, intraocular lens, vitreous, macula, and optic nerve (Kalogeropoulos et al. 2019; Naderan 2018). Previous reports indicate that intraocular pressure tends to decline during pregnancy, particularly as gestation progresses into later stages (Artunay et al. 2010).

At the molecular level, pregnancy-associated systemic adaptations are tightly regulated by epigenetic mechanisms. Recent evidence highlights the role of DNA methyltransferases in regulating trophoblast fusion and placental development, which are critical for maintaining maternal–fetal homeostasis. Altered DNA methylation patterns have been shown to influence vascular remodeling, immune tolerance, and metabolic signaling during pregnancy, thereby contributing to systemic physiological adaptations observed in gestation (Yang et al. 2024). These molecular regulatory processes provide a biological framework for understanding pregnancy-related changes in hematological and ocular physiology.

The assessment of hematological parameters provides valuable insight into overall animal health, as blood reflects systemic physiological status. The evaluation of blood parameters can assist in diagnosing disorders related to the hematopoietic system, general metabolic imbalances, and diseases of various systems and organs (Bezerra et al. 2017). Achieving sustainable livestock production in cattle farming requires enhancing reproductive potential without compromising animal welfare. Hematological evaluation is widely employed in dairy practice as a practical indicator of systemic health status (Hasan et al. 2021; Mekroud et al. 2021). By examining the hematological profile, it is possible to detect reproductive disorders in cows and to identify factors that influence biological markers such as disease, pregnancy or stress (Abramowicz et al. 2019; Loi et al. 2021; Noya et al. 2019; Purwar et al. 2019).

Despite the extensive body of research on bovine reproductive physiology, pregnancy-associated ocular changes remain poorly characterized. Considering the profound hormonal, vascular and immunological alterations that occur during gestation, the lack of bovine-specific data on ocular parameters constitutes a significant knowledge gap.

Pregnancy is also characterized by profound metabolic reprogramming aimed at supporting fetal growth and maternal adaptation. Recent studies focusing on reproductive physiology have demonstrated that altered lipid, glucose and amino acid metabolism is closely linked to hormonal fluctuations and immune regulation (Ruan et al. 2024). Such systemic metabolic shifts may indirectly influence hematological parameters and tissue-specific physiological responses, including ocular structures, emphasizing the importance of evaluating pregnancy-related changes within a holistic physiological context.

A review of the existing literature indicates that only a limited number of studies have assessed intraocular pressure and Schirmer tear test values in cows and to date, no studies conducted in Turkey have examined the impact of pregnancy on ocular parameters in Holstein cattle.

In this context, accurate ocular screening during pregnancy is clinically important to prevent misdiagnosis, safeguard animal welfare, and optimize herd health management. Therefore, the present study aims to evaluate the effects of advanced pregnancy on intraocular pressure, tear secretion and selected hemogram parameters in Holstein cows.

## Materials and methods

The present study was conducted on 30 clinically healthy Holstein cows, aged between 2 and 6 years (*n* = 30), housed on a private farm in the Tekirdağ province. Ethical approval was obtained from the Local Animal Ethics Committee of Tekirdağ Namık Kemal University. All animals underwent ultrasonographic examination (Hasvet WED-3100 V) and were allocated into two groups: cows in advanced pregnancy (*n* = 15) and non-pregnant cows (*n* = 15). The study was carried out during the spring season under natural photoperiod conditions. All animals were maintained under standardized housing, feeding, and reproductive management practices, and were kept in semi-open barns with natural daylight exposure.

**Fig. 1:**
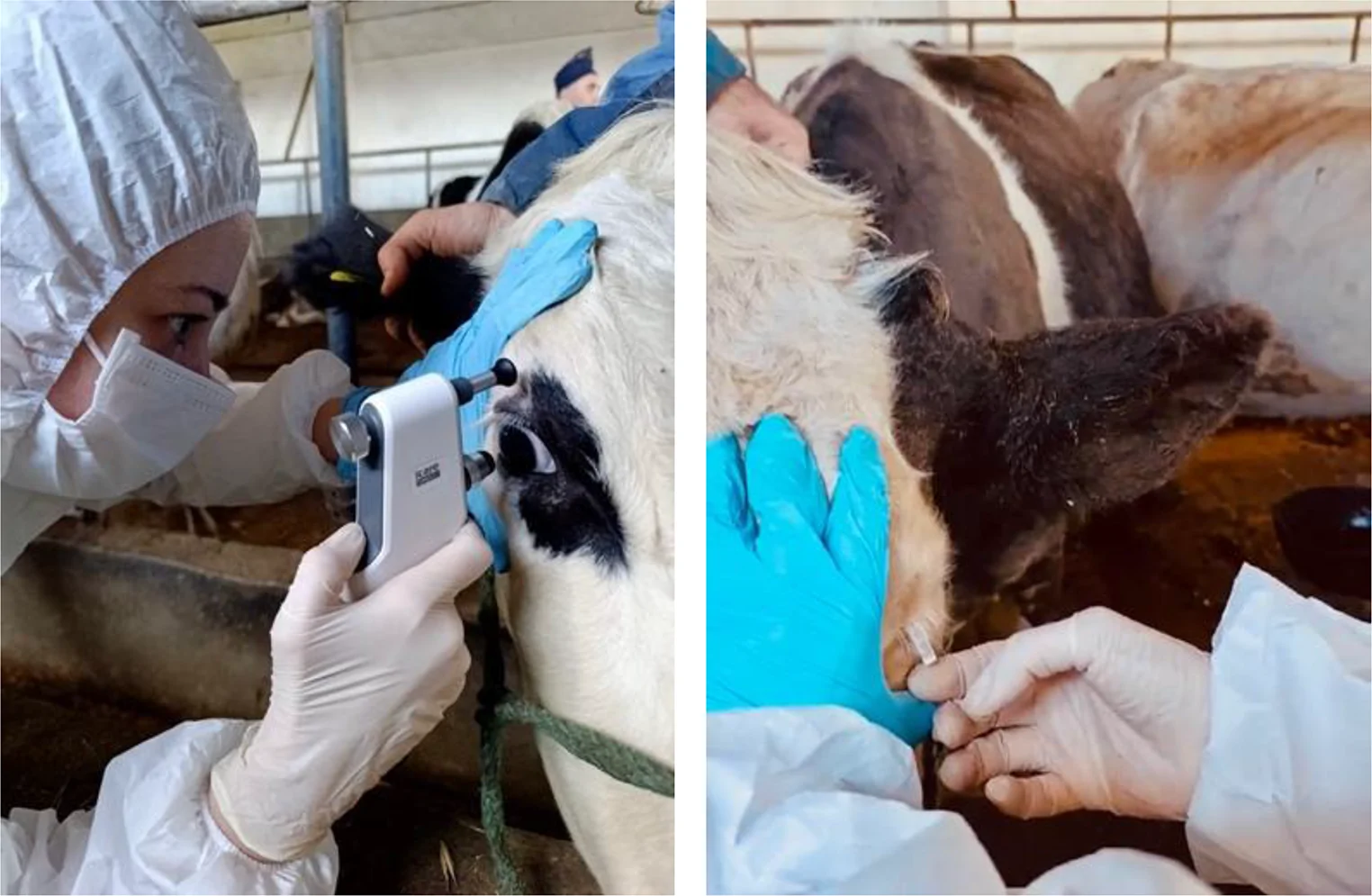
**a**, **b**. Measurement of IOP using the tonovet icare plus and measurement of STT

Immediately following pregnancy diagnosis, intraocular pressure (IOP) measurements were performed in both eyes of each animal using a Tonovet Icare Plus device. All ocular examinations were conducted in the morning hours (between 09:00 and 11:00) to minimize diurnal variations in IOP and tear secretion. Ambient temperature during measurements ranged between 18 and 22 °C. Tear secretion was subsequently assessed using Schirmer tear test strips, and the obtained values were recorded (Fig. 1a, and b). Following ocular examinations, blood samples were collected into EDTA-containing tubes for hematological analysis and promptly transported to the Veterinary Clinical Practice and Research Center Laboratory of Tekirdağ Namık Kemal University, where hemogram analyses were performed using an automated analyzer.

To minimize stress-related variability and operator bias, all ocular measurements were carried out by the same investigator within the animals’ housing environment. During the procedure, animals were gently restrained by a trained assistant using minimal manual head fixation for a short duration.

### Statistical analysis

Normality tests were first applied to the data obtained from the groups. For comparisons of intraocular pressure (IOP), Schirmer Tear Test (STT), and blood parameter test results between groups, the Independent Samples t-test was used for normally distributed paired groups, while the Mann–Whitney U test was applied for non-normally distributed data. A significance level of 5% (*p* = 0.05) was adopted for all statistical analyses.

## Results

According to the descriptive statistical results, the mean intraocular pressure (IOP) in pregnant cows was calculated as 33.07 mmHg, with a minimum value of 23 mmHg and a maximum of 50 mmHg. The mean value for the Schirmer Tear Test (STT) was 27.73 mm/min, with a minimum of 14 mm/min and a maximum of 35 mm/min (Table 1).

**Table 1:** Descriptive statistics of intraocular pressure (IOP) (mmHg) and Schirmer Tear Test (STT) (mm/min) results in pregnant and non-pregnant groups

Group	Test	Eyes	*n*	$\:\stackrel{-}{x}$	S	S$\:\stackrel{-}{x}$	Min	Max
Pregnant	IOP	Right	15	32.33	6.651	1.717	25	48
		Left	15	33.80	6.405	1.654	23	50
		Mean		33.07	6.458	1.179	23	50
	STT	Right	15	25.07	7.265	1.876	14	35
		Left	15	30.40	4.372	1.129	23	35
		Mean		27.73	6.486	1.184	14	35
Non-pregnant	IOP	Right	15	28.67	4.451	1.149	23	36
		Left	15	28.00	7.700	1.988	13	35
		Mean		29.80	4.759	0.869	23	41
	STT	Right	15	30.93	4.935	1.274	24	41
		Left	15	27.00	7.964	2.056	15	35
		Mean		27.50	7.714	1.408	13	35

In non-pregnant cows, the mean intraocular pressure was 29.80 mmHg, ranging from a minimum of 23 mmHg to a maximum of 41 mmHg. The mean STT value was 27.50 mm/min, with a minimum of 13 mm/min and a maximum of 35 mm/min.

Although a slight numerical difference was observed in intraocular pressure (IOP) values between the pregnant and non-pregnant groups, this difference was not statistically significant (*p* > 0.05; Table 2).

**Table 2:** Comparison of intraocular pressure (IOP) (mmHg) results between pregnant and non-pregnant groups

Group	*n*	Mean rank	Sum of ranks	U	*P*
Pregnant	15	34.73	1042.0	323.0	0.060
Non-pregnant	15	26.27	788.0		

*p* < 0.05: p=Mann Whitney U Test

Regarding STT values, no statistically significant difference was observed between the pregnant and non-pregnant groups (*p* > 0.05: Table 3).

**Table 3:** Comparison of Schirmer Tear Test (STT) (mm/min) results between pregnant and non-pregnant groups

Group	*n*	Mean rank	Sum of ranks	U	*P*
Pregnant	15	29.83	895.00	430.0	0.764
Non-pregnant	15	31.17	935.00		

*p* < 0.05: p=Mann Whitney U Test

No statistically significant difference was found between the intraocular pressure values of the right and left eyes in pregnant cows (*p* > 0.05: Table 4).

**Table 4:** Comparison of intraocular pressure (IOP) (mmHg) values between the right and left eyes of pregnant cows

Group	*n*	$\:\stackrel{-}{x}$	S$\:\stackrel{-}{x}$	*P*
Right eye	15	32.33	1.717	0.543
Left eye	15	33.80	1.654	

*p* < 0.05: p = Independent Samples t-Test

A statistically significant difference was observed between the Schirmer Tear Test results of the right and left eyes in pregnant cows (*p* < 0.05; Table 5).

**Table 5:** Comparison of Schirmer Tear Test (STT) (mm/min) results between the right and left eyes of pregnant cows

Group	*n*	$\:\stackrel{-}{x}$	S$\:\stackrel{-}{x}$	*P*
Right eye	15	25.07	1.876	0.021
Left eye	15	30.40	1.129	

*p* < 0.05: p = Independent Samples t-Test

No statistically significant difference was observed between the intraocular pressure values of the right and left eyes in non-pregnant cows (*p* > 0.05; Table 6).

**Table 6:** Comparison of Intraocular Pressure (IOP) (mmHg) results between the right and left eyes of non-pregnant cows

Group	*n*	Mean rank	Sum of ranks	U	*P*
Right eye	15	13.53	203.00	83.00	0.233
Left eye	15	17.47	262.00		

*p* < 0.05: p=Mann Whitney U Testi

No statistically significant difference was observed between the intraocular pressure values of the right and left eyes in non-pregnant cows (*p* > 0.05; Table 7).

**Table 7:** Comparison of Schirmer Tear Test (STT) (mm/min) results between the right and left eyes of non-pregnant animals

Group	*n*	Mean rank	Sum of ranks	U	*P*
Right eye	15	15.83	237.50	107.50	0.838
Left eye	15	15.17	227.50		

*p* < 0.05: p=Mann Whitney U Testi

### Hemogram Results

A statistically significant difference was observed only in MPV, %ALY and ALY values between pregnant and non-pregnant cows according to hemogram results (*p* < 0.05; Tables 8 and 9).

**Table 8:** Comparison of blood parameter results between pregnant and non-pregnant cows

Group	*n*	Parameters	$\:\stackrel{-}{x}$	S$\:\stackrel{-}{x}$	*P*
Pregnant	15	WBC	7.06	0.33	0.673
Non-pregnant	15		6.85	0.35	
Pregnant	15	%LYM	49.10	1.69	0.475
Non-pregnant	15		51.88	13.38	
Pregnant	15	%NEU	38.50	1.89	0.471
Non-pregnant	15		35.30	3.96	
Pregnant	15	LYM	3.46	0.20	0.786
Non-pregnant	15		3.57	0.32	
Pregnant	15	MON	0.67	0.04	0.391
Non-pregnant	15		0.75	0.07	
Pregnant	15	NEU	2.74	0.22	0.334
Non-pregnant	15		2.39	0.28	
Pregnant	15	RBC	5.96	0.12	0.059
Non-pregnant	15		6.54	0.27	
Pregnant	15	HGB	10.07	0.16	0.102
Non-pregnant	15		10.73	0.40	
Pregnant	15	HCT	29.67	0.49	0.077
Non-pregnant	15		32.20	1.30	
Pregnant	15	MCV	50.09	1.03	0.687
Non-pregnant	15		49.49	1.02	
Pregnant	15	MCH	16.83	0.36	0.538
Non-pregnant	15		16.47	0.45	

*p* < 0.05: p = Independent Samples t-Test

**Table 9:** Comparison of blood parameter results between pregnant and non-pregnant cows

Group	*n*	Parameters	Mean rank	Sum of ranks	U	*P*
Pregnant	15	%MON	13.93	209.00	89.00	0.330
Non-pregnant	15		17.07	256.00		
Pregnant	15	EOS	18.10	271.50	73.50	0.106
Non-pregnant	15		12.90	193.50		
Pregnant	15	BASO	14.57	218.50	98.50	0.557
Non-pregnant	15		16.43	246.50		
Pregnant	15	%EOS	17.80	267.00	78.00	0.152
Non-pregnant	15		13.20	198.00		
Pregnant	15	%BASO	14.47	217.00	97.00	0.515
Non-pregnant	15		16.53	248.00		
Pregnant	15	MCHC	16.80	252.00	93.00	0.418
Non-pregnant	15		14.20	213.00		
Pregnant	15	RDW_CV	15.13	227.00	107.00	0.819
Non-pregnant	15		15.87	238.00		
Pregnant	15	PLT	17.87	268.00	62.00	0.061
Non-pregnant	15		11.93	167.00		
Pregnant	15	MPV	19.93	299.00	31.00	0.001
Non-pregnant	15		9.71	136.00		
Pregnant	15	%ALY	11.17	167.50	47.50	0.007
Non-pregnant	15		19.83	297.50		
Pregnant	15	ALY	10.93	164.00	44.00	0.004
Non-pregnant	15		20.07	301.00		
Pregnant	15	%LIC	13.67	205.00	85.00	0.254
Non-pregnant	15		17.33	260.00		
Pregnant	15	LIC	13.70	205.50	85.50	0.262
Non-pregnant	15		17.30	259.50		
Pregnant	15	SMEAN(PLT)	18.60	279.00	66.00	0.054
Non-pregnant	15		12.40	186.00		
Pregnant	15	SMEAN(MPV)	20.93	314.00	31.00	0.001
Non-pregnant	15		10.07	151.00		

*p* < 0.05: p=Mann Whitney U Test

## Discussion

In this study, intraocular pressure (IOP), Schirmer Tear Test (STT) and various hemogram parameters were compared between pregnant and non-pregnant cows. Overall, gestation appeared to exert minimal influence on selected ocular measurements, whereas distinct alterations were evident in specific hematological indices.

Numerical differences were detected in IOP values between pregnant and non-pregnant cows; however, these differences were not statistically significant. This result is consistent with some studies in the literature. For example, Gebru et al. (2011) reported that pregnancy does not cause significant changes in intraocular pressure. In contrast, some human studies indicate that hormonal changes during pregnancy may reduce intraocular pressure (Qureshi et al. 1996). However, these effects may vary among species, and there is limited data on cattle.

Regarding the Schirmer Tear Test results, no significant difference was found between the pregnant and non-pregnant groups, suggesting that pregnancy does not affect lacrimation levels. However, a statistically significant difference was observed between the right and left eyes of pregnant cows. This finding is presented as a hypothesis and may reflect local physiological or anatomical variability rather than a definitive biological effect. Although bovine-specific data on interocular STT differences are limited, previous studies in cattle have reported wide ranges of normal STT values without significant interocular differences, while highlighting considerable variability in tear production among individuals and under different conditions (Tofflemire et al. 2015). Comparable interocular variability has also been described as part of normal physiological variation in human ophthalmologic studies (Gayton 2009).

When examining the hemogram parameters, significant differences were observed in MPV (Mean Platelet Volume), %ALY (Percentage of Atypical Lymphocytes) and ALY (Atypical Lymphocyte Count), between pregnant and non-pregnant cows. Elevated MPV may reflect physiological platelet activation during pregnancy. Indeed, various studies have documented increased platelet activity throughout pregnancy as a physiological adaptation to maintain hemostatic balance (Rath et al. 2015; Bozkurt et al. 2013).

Recent clinical evidence further supports the concept of immune modulation during reproductive processes. A prospective cohort study evaluating immune responses in couples undergoing assisted reproductive technologies demonstrated that controlled immune activation and lymphocyte profile modulation are essential components of reproductive success (Yang et al. 2025). These findings reinforce the interpretation that reduced %ALY and ALY values observed in pregnant cows may reflect physiological immune adaptation rather than pathological immune suppression.

Pregnancy is accompanied by controlled oxidative stress and adaptive antioxidant responses that support immune balance and tissue integrity. Experimental studies in animal models have shown that amino acids such as glutamate and aspartate play a protective role by enhancing antioxidant enzyme activity and supporting immune defense mechanisms (Tang et al. 2020). Although conducted in male reproductive physiology, these findings provide relevant insights into how systemic antioxidant and immune modulation may contribute to the physiological hematological adaptations observed during pregnancy.

Furthermore, lower %ALY and ALY values may indicate modulation of the immune system during pregnancy. Pregnancy induces immune tolerance by regulating immune cell populations. The literature describes changes in lymphocyte subgroups during pregnancy, including increased regulatory T cells and suppression of certain pro-inflammatory cells (Aluvihare et al. 2004).

Changes in iron metabolism during pregnancy may affect MCHC, but this effect may be less pronounced in cattle and requires further research in larger populations. Indeed, the current study did not find a statistically significant difference (Gül et al. 2010).

Limitations of this study include a relatively small sample size and lack of evaluation of hormonal variables. Additionally, ophthalmological parameters such as intraocular pressure and tear production may be influenced by seasonal and environmental factors.

## Conclusion

This study aimed to compare intraocular pressure (IOP), Schirmer Tear Test (STT), and various hematological parameters between pregnant and non-pregnant cows. The results showed no statistically significant differences between the groups regarding IOP and STT, except for a significant difference between the right and left eye Schirmer Test results in pregnant cows. This suggests that pregnancy may asymmetrically affect tear production between eyes, possibly due to local factors.

Hematological data indicated that pregnancy has statistically significant effects on certain blood parameters. Changes in MPV, %ALY, ALY, and MCHC values demonstrate that pregnancy modulates hematological responses. These differences can be associated with physiological immune adaptations and hematological dynamics during pregnancy.

The findings emphasize that pregnancy is a physiological state that should be considered during ophthalmological and hematological evaluations. Clinical examinations and laboratory assessments of pregnant animals should take these physiological changes into account, and pregnancy-related variations should not be mistaken for pathological conditions.

Recognizing physiological pregnancy-related variations in hematological and ocular parameters is clinically important, as it may help veterinarians avoid misinterpretation of normal gestational changes as pathological conditions, thereby reducing the risk of misdiagnosis in pregnant cows.

Future studies with larger sample sizes and longitudinal designs could better elucidate the temporal course of these physiological changes. Additionally, assessing the relationship between hormonal levels and these parameters would provide a more comprehensive understanding.

## Supplementary Information

Below is the link to the electronic supplementary material.


Supplementary Material 1


## Data Availability

The data that support the findings of this study are available on request from the corresponding author.
